# Vaccination of Rabbits with a Cholera Conjugate Vaccine Comprising O-Specific Polysaccharide and a Recombinant Fragment of Tetanus Toxin Heavy Chain Induces Protective Immune Responses against *Vibrio cholerae* O1

**DOI:** 10.4269/ajtmh.23-0259

**Published:** 2023-10-02

**Authors:** Meagan Kelly, Suhi Jeon, Jeesun Yun, Byungman Lee, Minchul Park, Yoonhee Whang, Chankyu Lee, Richelle C. Charles, Taufiqur R. Bhuiyan, Firdausi Qadri, Mohammad Kamruzzaman, Somyoung Cho, Willie F. Vann, Peng Xu, Pavol Kováč, Ravi Ganapathy, Julia Lynch, Edward T. Ryan

**Affiliations:** ^1^ Division of Infectious Diseases, Massachusetts General Hospital, Boston, Massachusetts;; ^2^ Eubiologics Ltd, Gangnam-gu, Seoul, South Korea;; ^3^ Department of Biological Engineering, Inha University, Incheon, South Korea;; ^4^ Department of Medicine, Harvard Medical School, Boston, Massachusetts;; ^5^ Department of Immunology and Infectious Diseases, Harvard T. H. Chan School of Public Health, Boston, Massachusetts;; ^6^ icddr,b (International Centre for Diarrhoeal Disease Research, Bangladesh), Dhaka, Bangladesh;; ^7^ International Vaccine Institute, Seoul, South Korea;; ^8^ Center for Biologics Evaluation and Research, U.S. Food and Drug Administration, Silver Spring, Maryland;; ^9^ National Institute of Diabetes and Digestive and Kidney Diseases, Laboratory of Bioorganic Chemistry, NIH, Bethesda, Maryland

## Abstract

There is a need for next-generation cholera vaccines that provide high-level and durable protection in young children in cholera-endemic areas. A cholera conjugate vaccine (CCV) is in development to address this need. This vaccine contains the O-specific polysaccharide (OSP) of *Vibrio cholerae* O1 conjugated via squaric acid chemistry to a recombinant fragment of the tetanus toxin heavy chain (OSP:rTTHc). This vaccine has been shown previously to be immunogenic and protective in mice and found to be safe in a recent preclinical toxicological analysis in rabbits. We took advantage of excess serum samples collected as part of the toxicological study and assessed the immunogenicity of CCV OSP:rTTHc in rabbits. We found that vaccination with CCV induced OSP-, lipopolysaccharide (LPS)-, and rTTHc-specific immune responses in rabbits, that immune responses were functional as assessed by vibriocidal activity, and that immune responses were protective against death in an established virulent challenge assay. CCV OSP:rTTHc immunogenicity in two animal model systems (mice and rabbits) is encouraging and supports further development of this vaccine for evaluation in humans.

## INTRODUCTION

Currently available oral cholera vaccines (OCVs) are integral to global strategies to control cholera. Unfortunately, currently available killed OCVs provide short-term and lower-level protection in young children compared to that in adults and older children in endemic areas.^1^^–^^5^ This is problematic in endemic areas where cholera disproportionally affects young children.^6^^,^^7^ In such areas, children younger than 5 years of age have a 2 to 4 times higher incidence rate of cholera than those in the general population.^6^^,^^8^^,^^9^ Thus, there is a need for next-generation cholera vaccines that provide high-level and durable immunity in such young children. Immunity protective against cholera following infection or vaccination targets the O-specific polysaccharide (OSP) of *Vibrio cholerae*^10^^–^^12^ Unfortunately, young children do not develop prominent immune responses to polysaccharides, including OSP, which are T cell-independent antigens. Conjugating polysaccharides to protein carriers can induce T cell-dependent immune responses in young children.^13^^–^^18^ We have therefore developed a cholera conjugate vaccine (CCV) comprised of OSP of *V. cholerae* O1 Inaba conjugated to a recombinant fragment of tetanus toxin heavy chain (rTTHc) because Inaba provides protection against both Inaba and Ogawa serotypes of *V. cholerae* O1.^19^ We have previously characterized this vaccine and described its development and production in detail and have shown it to be protectively immunogenic in mice^19^^,^^20^ As part of next-stage assessment of CCV OSP:rTTHc and in support of regulatory filing for phase 1 evaluation of CCV in humans, we next performed a toxicological analysis of CCV in animals, including rabbits. We took advantage of excess serum samples collected from rabbits in these studies and performed a secondary immunogenicity analysis, assessing induction of OSP-, LPS-, and rTTHc-specific antibody responses, the ability of vaccination to induce vibriocidal responses, and the ability of immune sera to protect against virulent challenge in an established model.

## MATERIALS AND METHODS

Cholera conjugate vaccine was produced and characterized by Eubiologics Co., Ltd. (Gangnam-gu, Seoul, South Korea) as previously described.^19^ Toxicology studies were performed at Eurofins Advinus Limited (Bengaluru, India). Immunologic analysis was performed at the Massachusetts General Hospital (Boston, MA, USA).

In the toxicological assessment of CCV, New Zealand White rabbits (*Oryctolagus cuniculus*) received three intramuscular injections (0.5 mL/inoculation, thigh) of CCV or placebo with or without aluminum phosphate (on days 1, 15, and 29). In total, 20 male and 20 female rabbits were randomized into 4 groups (5/sex/group: placebo [buffer plus excipients, with/without alum] or test article [CCV, 40 µg based on polysaccharide component] in buffer plus excipients, with/without alum) and sacrificed on day 31, 2 days after the third vaccination on day 29. In a separate recovery cohort, 12 male and 12 female rabbits were randomized into four groups (3/sex/group: placebo [buffer plus excipients, with/without alum] or test article [CCV, 40 µg based on polysaccharide component] in buffer plus excipients, with/without alum) and were sacrificed on day 44, 15 days after the third dose on day 29. Blood was collected from all animals in labeled tubes without anticoagulant from the medial ear artery. Samples were kept at room temperature for at least 20 minutes prior to centrifugation. Serum was separated in a refrigerated centrifuge at 5,000 rpm for 5 minutes and stored at ≤ −70°C. Immunogenicity analysis used excess serum samples from day 1 (prevaccination) and at time of sacrifice (day 31 or 44).

### Antigen-specific antibody responses in serum.

We assessed OSP-specific and LPS-specific IgG and IgM responses and rTTHc-specific IgG responses in serum using kinetic ELISAs as previously described.^19^ O-specific polysaccharide and LPS were purified from *V. cholerae* O1 El Tor strain PIC018 as previously described.^19^ Briefly, 96-well Nunc plates (Thermo Fisher, Grand Island, NY) were coated with 100 ng/well of OSP conjugated to bovine serum albumin (OSP:BSA) or rTTHc or with 25 ng/well of LPS in 50 mM carbonate buffer, pH 9.6. Diluted sera were added in duplicate to plates (1:2,500 IgG anti-OSP and anti-rTTHc, 1:250 IgG anti-LPS, 1:25 IgM anti-OSP and anti-LPS) and bound antibodies were detected using horseradish peroxidase conjugated to anti-rabbit IgG antibody (Rockland Immunochemicals, Pottstown, PA) or anti-rabbit IgM antibody (LSBio, Shirley, MA) (diluted 1:20,000). Peroxidase activity was measured at 405 nm using the chromogenic substrate 2,2′-azinobis(3-ethylbenzothiazoline-6-sulfonic acid) (ABTS; Sigma, Burlington, MA). Plates were read for 5 minutes at 30-second intervals, and the maximum slope for an optical density change of 0.2 U was reported as millioptical density units per minute (mOD/min). To establish the immunoreactivity of individual samples, mOD/min values were divided by the geometric mean of four plate control wells containing purified rabbit IgG or purified rabbit IgM (100 ng/well; Southern Biotech, Birmingham, AL), and the obtained values were then multiplied by 100 and reported as ELISA units (EU). Following vaccination, a responder was defined as having more than a 3-fold increase (OSP, rTTHc IgG) or a 4-fold increase (LPS IgG) of ELISA units compared with the matched prevaccination value.

### Vibriocidal responses.

We assessed serum vibriocidal antibody titers against *V. cholerae* O1 El Tor Inaba strain PIC018 as previously described.^19^^–^^21^ The vibriocidal titer was calculated as the dilution of serum causing a 50% reduction in optical density compared with that of control wells without serum. We defined responders as having at least a 4-fold increase in vibriocidal titer compared with the prevaccination value.

### Protection assays.

To assess protection afforded by vaccination, we used the standard cholera neonatal mouse cholera challenge assay, as previously described,^19^^,^^20^ using wild-type *V*. *cholerae* O1 El Tor Inaba strain N16961 as the challenge strain. In brief, we removed 3- to 5-day-old unimmunized CD-1 suckling mice from dams 2 hours prior to inoculation. We then administered to pups a 50-μL inoculum comprised of 6 × 10^9^ colony-forming units (CFU) of *V*. *cholerae* O1 El Tor Inaba strain N16961 mixed with a 1:1 dilution of pooled terminal serum from rabbits intramuscularly immunized with placebo (buffer, excipients, aluminum phosphate), CCV OSP:rTTHc, or CCV OSP:rTTHc with aluminum phosphate. Following oral challenge, we kept neonates at 30°C and monitored animals every 3 hours for 36 hours, after which surviving animals were euthanized.

### Statistics and graphs.

Data were compared within groups across time points using Wilcoxon signed-rank tests, and across groups using Mann-Whitney *U* tests. Response rates were compared using χ^2^ tests. We performed statistical analyses using GraphPad Prism 7 (GraphPad Software, Inc., San Diego, CA).

## RESULTS

**Figure 1.**
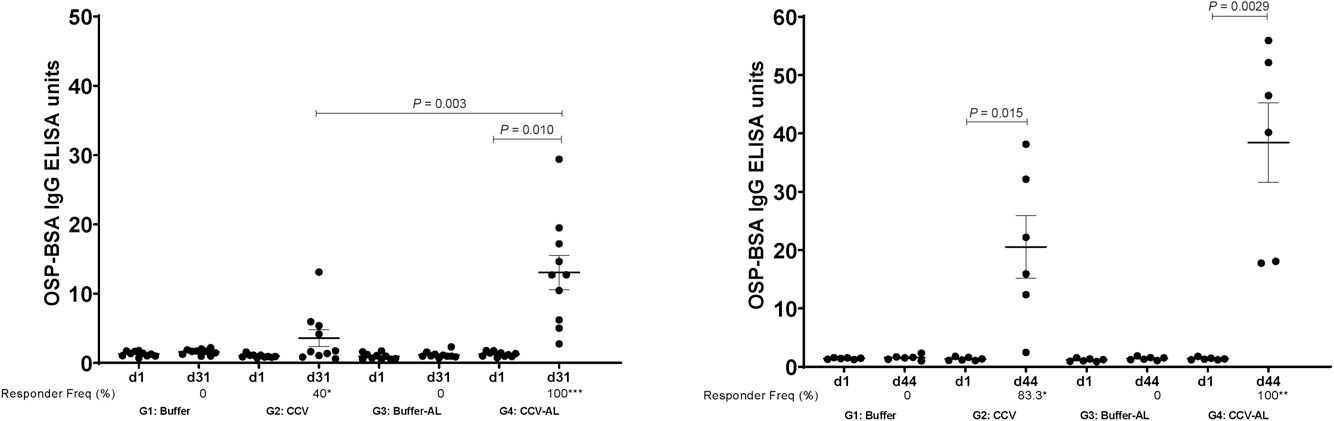
Serum IgG responses in rabbits at listed time points against *V. cholerae* O1 O-specific polysaccharide (OSP) in various vaccine cohorts (groups 1 to 4 [G1 to G4]) after intramuscular immunization with buffer or cholera conjugate vaccine (CCV) on days 1, 15, and 29, with or without the adjuvant alum (AL). Dots represent responses in individual animals. The mean and standard error of the mean are shown. Statistically significant differences among compared cohorts are represented (mean or responder frequencies). Responder frequencies are also listed on the *x* axis, with an asterisk denoting a statistically significant increase in responder frequency from day 1 (d1) to sacrifice on day 31 (d31) or day 44 (d44) (*P* < 0.05); ** *P* ≤ 0.01; *** *P* ≤ 0.001. (See Supplemental Table 1 for details.)

**Figure 2.**
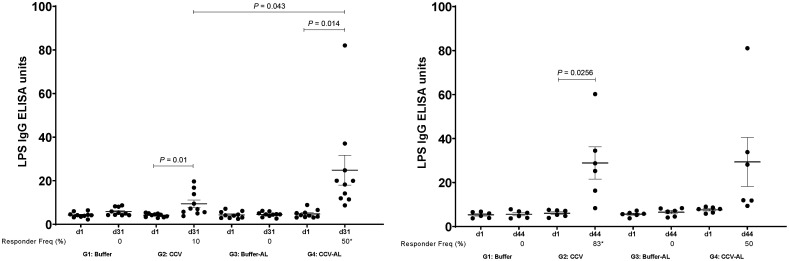
Serum IgG responses in rabbits at listed time points against *V. cholerae* O1 lipopolysaccharide (LPS) in various vaccine cohorts (groups 1 to 4 [G1 to G4]) following intramuscular immunization with buffer or cholera conjugate vaccine (CCV) on days 1, 15, and 29, with or without the adjuvant alum (AL). Dots represent responses in individual animals. The mean and standard error of the mean are shown. Statistically significant differences among compared cohorts are represented (mean or responder frequencies). Responder frequencies are also listed on the *x* axis, with an asterisk denoting a statistically significant increase in responder frequency from day 1 (d1) to sacrifice on day 31 (d31) or day 44 (d44) (*P* < 0.05); ** *P* ≤ 0.01; *** *P* ≤ 0.001. (See Supplemental Table 2 for details.)

**Figure 3.**
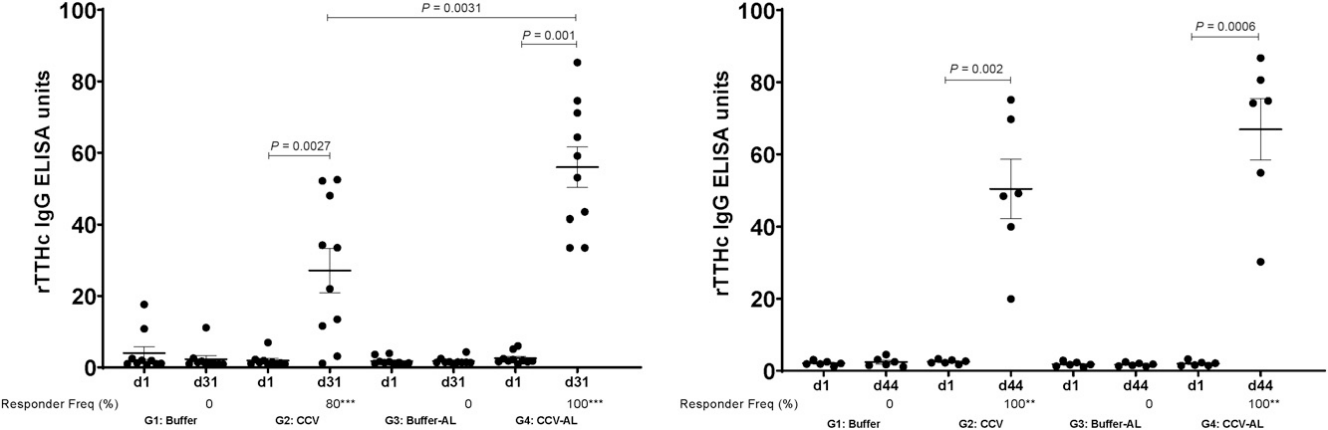
Serum IgG responses in rabbits at listed time points against the recombinant fragment of the tetanus toxin heavy chain (rTTHc) in various vaccine cohorts (groups 1 to 4 [G1 to G4]) following intramuscular immunization with buffer or cholera conjugate vaccine (CCV) on days 1, 15, and 29, with or without the adjuvant alum (AL). Dots represent responses in individual animals. The mean and standard error of the mean are shown. Statistically significant differences among compared cohorts are represented (mean or responder frequencies). Responder frequencies are also listed on the *x* axis, with an asterisk denoting a statistically significant increase in responder frequency from day 1 (d1) to sacrifice on day 31 (d31) or day 44 (d44) (*P* < 0.05); ** *P* ≤ 0.01; *** *P* ≤ 0.001. (See Supplemental Table 3 for details.)

**Figure 4.**
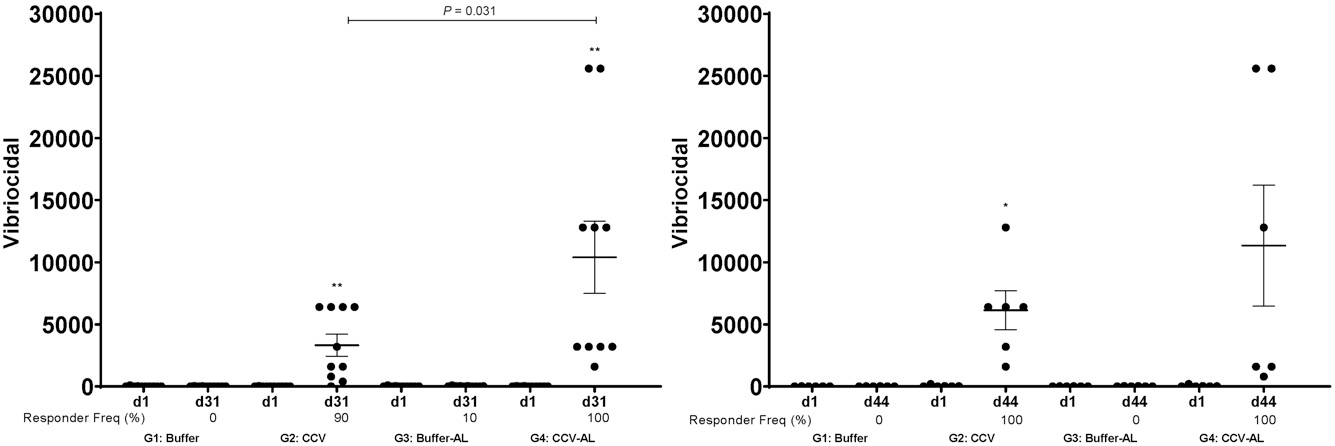
Vibriocidal responses in vaccinated cohorts (groups 1 to 4 [G1 to G4]) of mice following intramuscular immunization with buffer or cholera conjugate vaccine (CCV) on days 1, 15 and 29, with or without the adjuvant alum (AL). Dots represent responses in individual rabbits on day 1 (d1) and day 31 (d31) or day 44 (d44). The mean and standard error of the mean are reported for each group. Comparison of d1 to d33 or d44 responses: * *P* ≤ 0.05; ** *P* ≤ 0.01

**Figure 5.**
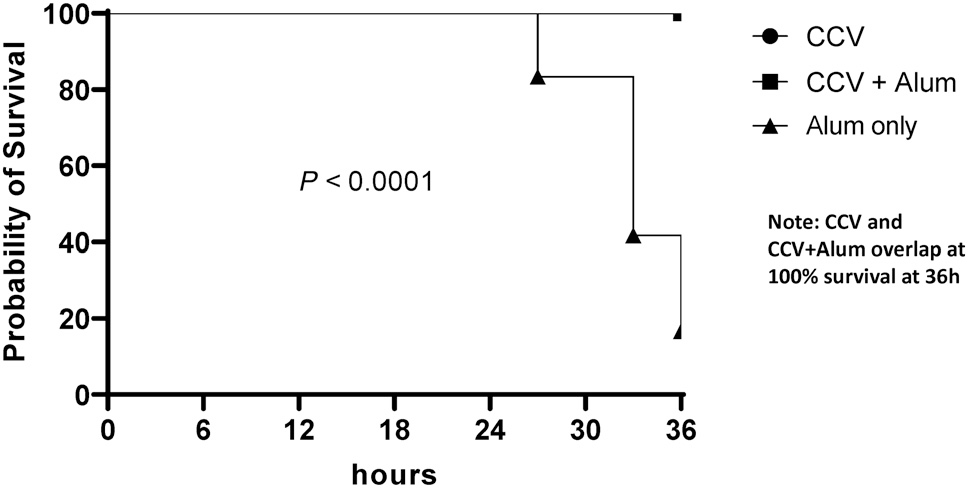
Survival curves of experimentally challenged animals by cohort. Three- to five-day-old CD-1 mouse pups were administered by oral gavage 50 μL of a preparation containing 6 × 10^9^ colony-forming units of wild-type *V*. *cholerae* N16961 mixed with a 1:1 dilution of pooled terminal serum from rabbits intramuscularly immunized with alum alone (CD-1 cohort, *N =* 12) or conjugate vaccine (O-specific polysaccharide:recombinant fragment of the tetanus toxin heavy chain [OSP:rTTHc]) with (CD-1 cohort, *N =* 10) or without (CD-1 cohort, *N =* 12) immunoadjuvantative alum. CCV and CCV+Alum data overlap in the figure because both maintained 100% survival throughout the assessment.

Vaccination with CCV induced significant OSP-specific IgG responses in animals by the time of sacrifice. Both magnitude of response (Figure 1) and responder frequency (Supplemental Table 1) were increased in the presence of alum, and responses were higher in samples collected 15 days after last vaccination than 48 hours after last vaccination. Low but significant levels of IgM OSP-specific responses were also detected at the time of sacrifice (Supplemental Figure 1). Similarly, vaccination also induced significant LPS-specific IgG (Figure 2, Supplemental Table 2) and IgM (Supplemental Figure 2) immune responses and rTTHc-specific IgG (Figure 3, Supplemental Table 3) responses. Based on our previous analysis in mice, we did not assess IgA responses. Significant vibriocidal responses were evident in postvaccination samples (Figure 4), and sera from vaccinated rabbits conferred protection in the murine virulent challenge assay, with 100% survival associated with sera from rabbits vaccinated with CCV with or without alum and 17% survival associated with sera from rabbits vaccinated with alum alone (Figure 5; *P* < 0.001).

## DISCUSSION

We took advantage of having access to excess serum samples from a toxicological analysis in rabbits in which CCV appeared safe to further evaluate CCV immunogenicity in a second animal model. Our results demonstrate that CCV OSP:rTTHc is immunogenic in rabbits, as we previously found in mice.^19^^,^^20^ Vaccination of rabbits induce vibriocidal and OSP antibody responses immune responses that are associated with protection against cholera in humans^10^^–^^12^; and postvaccination sera afforded protection in an established virulent challenge assay. Protection against cholera is serogroup specific,^22^^–^^24^ and serogroup specificity is defined by the OSP component of LPS. OSP-specific responses have been associated with protection in human experimental challenge models of cholera,^10^^,^^25^ as well as in household contact studies in cholera-endemic areas.^11^^,^^25^ The vibriocidal response uses a functional assay, and although vibriocidal responses are associated with protection against cholera, they are not thought to be mechanistic but, instead, a surrogate marker of protection at the mucosal surface and have been shown to largely target *V. cholerae* OSP.^12^^,^^19^^,^^26^^–^^28^

Because our analysis involved the secondary use of excess samples collected for a nonimmunological study, we were limited in our analysis to a comparison of prevaccination immune responses versus those at the time of sacrifice. We can thus not ascertain whether the significantly higher immune responses in the cohort of animals killed 15 days after last (third) vaccination compared with those killed 2 days after last (third) vaccination represent the impact of the third dose or maturation of immune responses that were already in process. We can also not comment on the induction of immune responses after just one or two doses of vaccine in rabbits. The three-dose toxicology study was designed to test the vaccine at more doses than anticipated for possible use in humans (one or two vaccinations). Similarly, the toxicology study was designed to test the vaccine at a dose (40 µg of polysaccharide per vaccine dose) that exceeded the anticipated future dose in humans (5 to 25 µg of polysaccharide per vaccine dose). We cannot therefore comment on the immunogenicity of the vaccine at lower doses in rabbits. However, we previously analyzed dose ranges in mice and found comparable immunogenicity across the range of 10 to 50 µg of polysaccharide per dose^20^; we also found that immune responses were detectable following a single dose of vaccine in mice, including at the 10-µg polysaccharide dosing.^19^^,^^20^ Our rabbit-based study did, however, allow us the ability to directly assess the impact of immunoadjuvantative alum on immunogenicity, and our target immune responses were significantly higher in the presence of aluminum phosphate than with no alum in rabbits. This is similar to what we previously observed in mice.^19^ In a comparison of immune responses across species, rTTHc-specific antibody responses were found comparable in magnitude in rabbits and mice; however, OSP-specific, LPS-specific, and vibriocidal responses were approximately 10^2^ to 10^3^ higher in rabbits than those observed in mice after comparable vaccination, suggesting that different species can respond differently to the same vaccine and underscoring the utility of using more than one animal model in preclinical evaluation.^19^^,^^20^ We also did not directly assess immune responses or protection against *V. cholerae* O1 Ogawa serotype organisms, although Inaba and Ogawa responses correlate highly.

Immune responses protective against cholera need to work at the mucosal surface since *V. cholerae* is a noninvasive enteric pathogen. Although mucosal immunization with currently available OCVs can induce mucosal IgM and IgA responses, these responses tend to be relatively short-lived, and young children respond poorly to polysaccharide antigens in comparison with older children and adults. A meta-analysis suggests a two-dose efficacy of 58% overall for currently available killed OCVs, with 64% in individuals older than 5 years of age and only 30% in children younger than 5 years of age.^7^ Vaccine efficacy is noted to be 55% to 60% within the first 2 years of OCV vaccination but falls to 39% in year 3 and 26% in year 4.^7^ The lower efficacy in young children has been one reason that OCVs have not been incorporated into the Expanded Program on Immunization in low- and middle-income settings. OCVs are now administered largely through one-off single regional campaigns, either preventively or in reaction to a cholera outbreak. However, the relatively short duration of protection afforded by OCVs suggests that repetitive vaccine campaigns would usually be required for sustained control of cholera, and the ongoing need for resources and the impact of competing public health priorities will in all likelihood limit the feasibility and practicality of such an approach.

It is within this context that next-generation cholera vaccines are needed. This is especially apparent in cholera-endemic areas where young children bear a large burden of cholera. The area in South Asia at the top of the Bay of Bengal is the historic home for cholera,^29^ and molecular epidemiologic analysis suggests that this region has been associated with the recurrent waves of cholera that have occurred throughout the world over the last few decades.^30^ A reasonable case can thus be made that a successful global cholera control strategy will require control of cholera in this region, and in this highly endemic region, protecting young children from cholera will be required. To address this need, we have thus developed a CCV for use in young children. Conjugate vaccines targeting mucosal pathogens have previously been found to be highly effective in young children, not only in preventing invasive disease but also in decreasing mucosal colonization and carriage of serotypes included in the conjugate vaccine, suggesting mucosal effector functions.^31^^–^^36^ In our analysis, low-level IgM responses against OSP and LPS were detectable following vaccination, although their duration and significance is uncertain. As expected, the primary immune response following vaccination with CCV was induction of antigen-specific IgG responses. Conjugate vaccines largely induce IgG responses, and although they are poor inducers of IgA and IgM, parenteral vaccination with conjugates does induce anti-polysaccharide IgG that appears in mucosal fluids, even in young children, but not following vaccination with unconjugated polysaccharide.^37^^–^^42^ Cholera is also particularly common among populations with tropical-environmental enteropathy, mucosal breakdown, and disruption of intestinal integrity, suggesting that exudative leakage of IgG antibodies might also contribute to protection in such areas.^43^^,^^44^ Indeed, previous nonconjugated parenteral cholera vaccines induced 40% to 70% protection against cholera, including in young children in cholera-endemic areas, but historical parenteral cholera vaccines were eventually abandoned in the 1980s as mucosal vaccine approaches gained prominence and public health efforts shifted to intense advancement of improved water and sanitation.^45^^–^^51^ Although such water and sanitary efforts are critical to long-term control of cholera and many other intestinal microbial diseases, the reality is that it will be decades before the global population most at risk of cholera will have access to safe water and adequate sanitation sufficient to eliminate cholera, and thus there is an urgent need for next-generation cholera vaccines to assist public health efforts.

In conclusion, building upon our previous analysis in mice, we have demonstrated here that a next-generation CCV in development is immunogenic in a second animal model (rabbits) and that these responses are functional and protective in established models. The CCV appeared safe in preclinical toxicologic analysis is currently undergoing phase 1 testing in humans (ClinicalTrials.gov number NCT05559983).

## Supplemental Materials


Supplemental materials
