# Supplementary material for: Vaccination of Rabbits with a Cholera Conjugate Vaccine Comprising O-Specific Polysaccharide and a Recombinant Fragment of Tetanus Toxin Heavy Chain Induces Protective Immune Responses against Vibrio cholerae O1
Source: Am J Trop Med Hyg. 2023 Oct 2;109(5):1122–8. doi: 10.4269/ajtmh.23-0259 (PMC10622467; doi:10.4269/ajtmh.23-0259)
Supplement: Supplementary file 1 [file tpmd230259.SD1.pdf]

Responder Frequency (OSP IgG)

N = 10 each cohort (D31), 6 each (D44)

Rabbit 1:2500

G1: Buffer D1	G1: Buffer D31	Chi2
0	0	N/A

G1: Buffer D1	G1: Buffer D44	Chi2
0	0	N/A

G2: CCV D1	G2: CCV D31	Chi2
0	4	0.0253

G2: CCV D1	G2: CCV D44	Chi2
0	5	0.0152

G3: Buffer-AL D1	G3: Buffer-AL D31	Chi2
0	0	N/A

G3: Buffer-AL D1	G3: Buffer-AL D44	Chi2
0	0	N/A

G4: CCV-AL D1	G4: CCV-AL D31	Chi2
0	10	<0.0001

G4: CCV-AL D1	G4: CCV-AL D44	Chi2
0	6	0.0022

G1: Buffer D31	G2: CCV D31	Chi2
0	0	N/A

G1: Buffer D44	G2: CCV D44	Chi2
0	5	0.0152

G3: Buffer-AL D31	G4: CCV-AL D31	Chi2
0	10	<0.0004

G3: Buffer-AL D44	G4: CCV-AL D44	Chi2
0	6	0.0022

G2: CCV D31	G4: CCV-AL D31	Chi2
4	10	0.0034

G2: CCV D31	G4: CCV-AL D31	Chi2
5	6	1

Supplemental Table 1: Responder Frequency OSP IgG listed comparing vaccine cohorts based on antibody level on day 1 versus day 31/44. Cohorts received either buffer alone, CCV alone, CCV with Alum, or Buffer-Alum alone (as described in text).

Responder Frequency (LPS IgG)

N = 10 each cohort (D31), 6 each (D44)

Rabbit 1:250

G1: Buffer D1	G1: Buffer D31	Chi2
0	0	N/A

G1: Buffer D1	G1: Buffer D44	Chi2
0	0	N/A

G2: CCV D1	G2: CCV D31	Chi2
0	1	1

G2: CCV D1	G2: CCV D44	Chi2
0	5	0.0152

G3: Buffer-AL D1	G3: Buffer-AL D31	Chi2
0	0	N/A

G3: Buffer-AL D1	G3: Buffer-AL D44	Chi2
0	0	N/A

G4: CCV-AL D1	G4: CCV-AL D31	Chi2
0	5	0.0325

G4: CCV-AL D1	G4: CCV-AL D44	Chi2
0	3	0.1818

G1: Buffer D31	G2: CCV D31	Chi2
0	1	N/A

G1: Buffer D44	G2: CCV D44	Chi2
0	5	0.0152

G3: Buffer-AL D31	G4: CCV-AL D31	Chi2
0	5	0.0325

G3: Buffer-AL D44	G4: CCV-AL D44	Chi2
0	3	0.1818

G2: CCV D31	G4: CCV-AL D31	Chi2
1	5	0.1409

G2: CCV D31	G4: CCV-AL D31	Chi2
5	3	0.5455

Supplemental Table 2: Responder Frequency LPS IgG listed comparing vaccine cohorts based on antibody level on day 1 versus day 31/44. Cohorts received either buffer alone, CCV alone, CCV with Alum, or Buffer-Alum alone (as described in text).

Responder Frequency (rTTHc IgG)

N = 10 each cohort (D31), 6 each (D44)

Rabbit 1:2500

G1: Buffer D1	G1: Buffer D31	Chi2
0	0	N/A

G1: Buffer D1	G1: Buffer D44	Chi2
0	0	N/A

G2: CCV D1	G2: CCV D31	Chi2
0	8	0.0007

G2: CCV D1	G2: CCV D44	Chi2
0	6	0.0022

G3: Buffer-AL D1	G3: Buffer-AL D31	Chi2
0	0	N/A

G3: Buffer-AL D1	G3: Buffer-AL D44	Chi2
0	0	N/A

G4: CCV-AL D1	G4: CCV-AL D31	Chi2
0	10	<0.0001

G4: CCV-AL D1	G4: CCV-AL D44	Chi2
0	6	0.0022

G1: Buffer D31	G2: CCV D31	Chi2
0	8	0.0007

G1: Buffer D44	G2: CCV D44	Chi2
0	6	0.0022

G3: Buffer-AL D31	G4: CCV-AL D31	Chi2
0	10	<0.0001

G3: Buffer-AL D44	G4: CCV-AL D44	Chi2
0	6	0.0022

G2: CCV D31	G4: CCV-AL D31	Chi2
8	10	0.4737

G2: CCV D31	G4: CCV-AL D31	Chi2
6	6	1

Supplemental Table 3: Responder Frequency rTTHc IgG listed comparing vaccine cohorts based on antibody level on day 1 versus day 31/44. Cohorts received either buffer alone, CCV alone, CCV with Alum, or Buffer-Alum alone (as described in text).

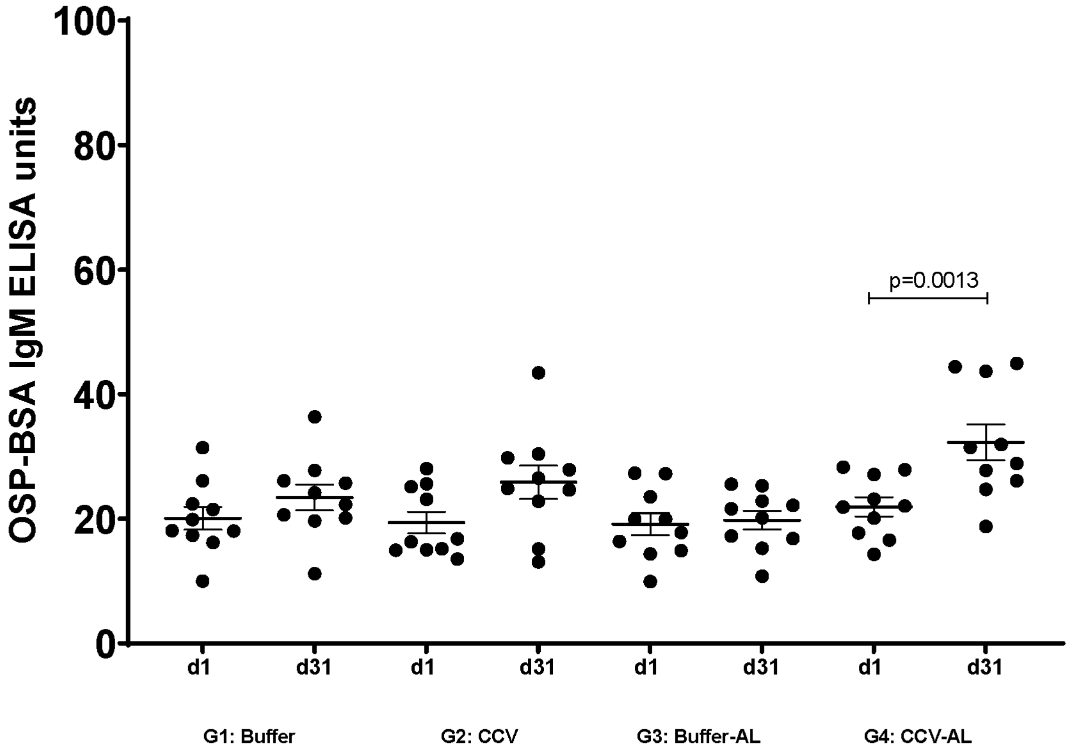

OSP-BSA IgM ELISA units
100
80
60
40
20
0
p=0.0013
d1
d31
d1
d31
d1
d31
d1
d31
G1: Buffer
G2: CCV
G3: Buffer-AL
G4: CCV-AL

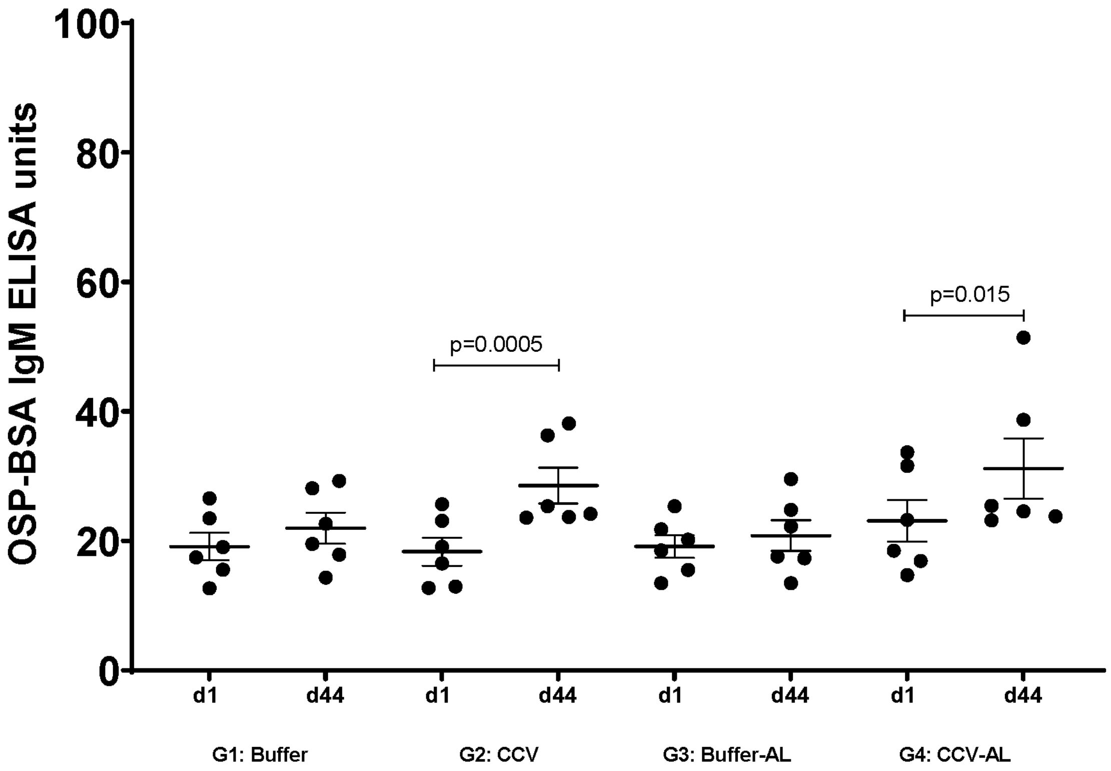

OSP-BSA IgM ELISA units
100
80
60
40
20
0
p=0.0005
p=0.015
d1
d44
d1
d44
d1
d44
d1
d44
G1: Buffer
G2: CCV
G3: Buffer-AL
G4: CCV-AL

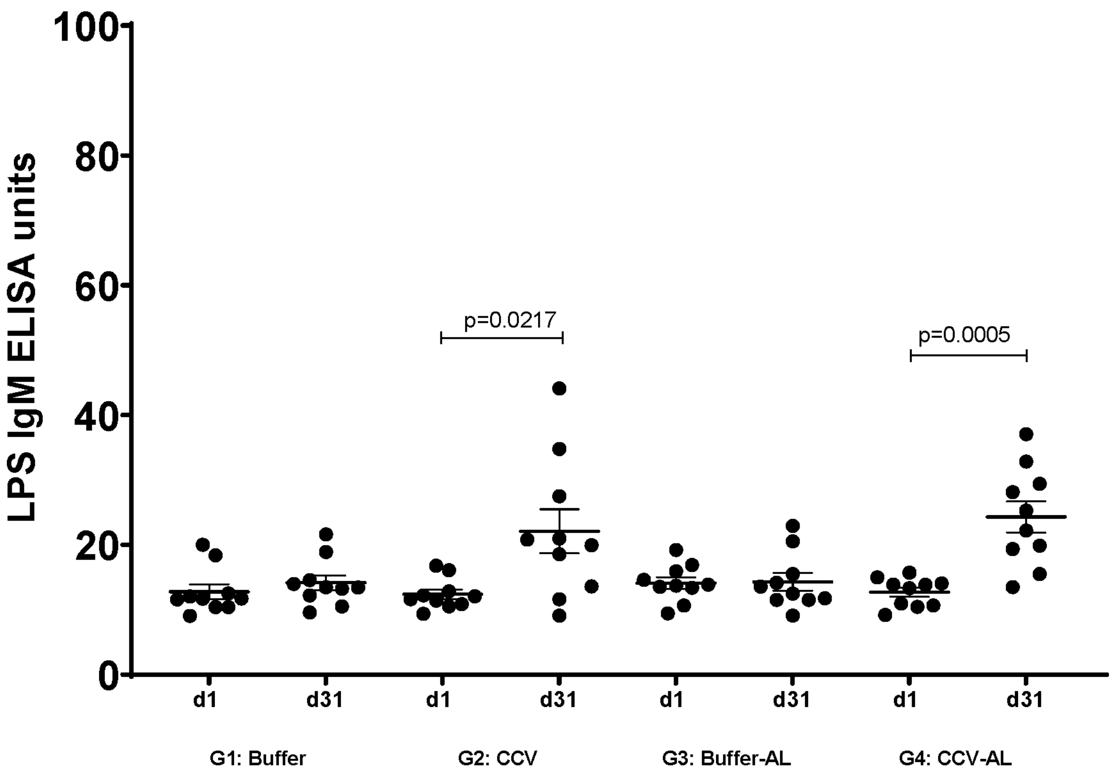
100
80
60
40
20
0
LPS IgM ELISA units
p=0.0217
p=0.0005
d1
d31
d1
d31
d1
d31
d1
d31
G1: Buffer
G2: CCV
G3: Buffer-AL
G4: CCV-AL

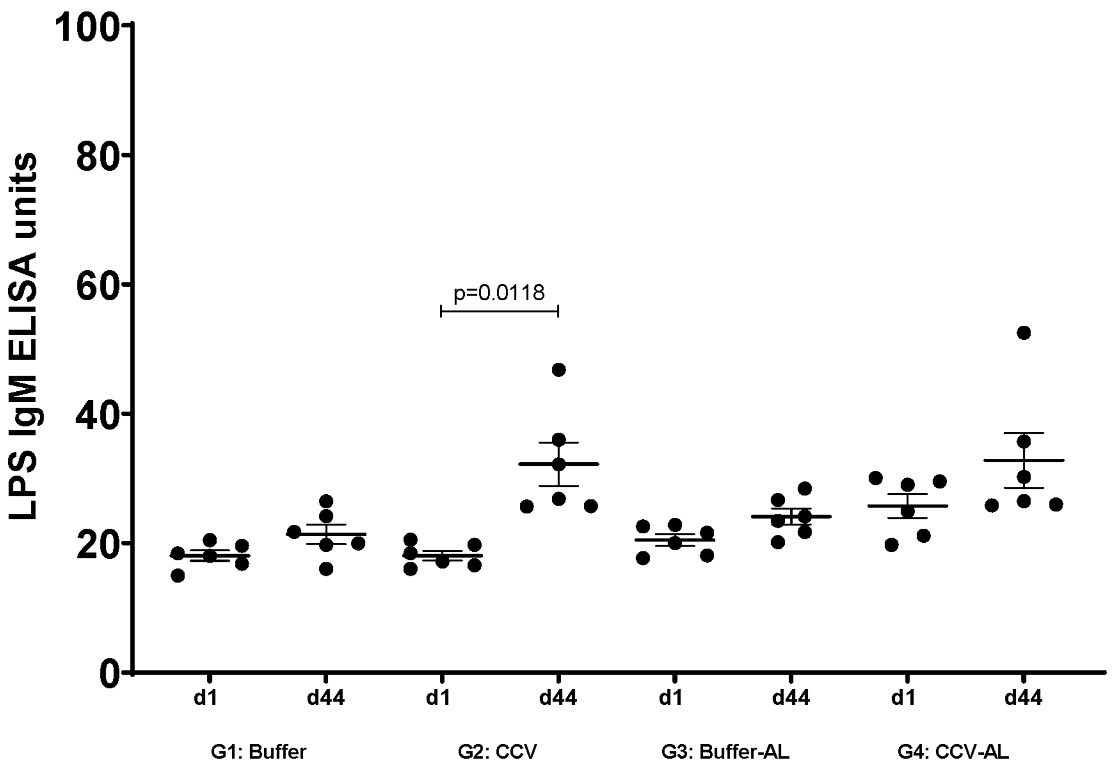
100
80
60
40
20
0
LPS IgM ELISA units
p=0.0118
d1
d44
d1
d44
d1
d44
d1
d44
G1: Buffer
G2: CCV
G3: Buffer-AL
G4: CCV-AL
